# Complete Genome Sequence of Mirabilis Crinkle Mosaic Virus Isolated from Pokeweed in Japan

**DOI:** 10.1128/MRA.00283-21

**Published:** 2021-05-27

**Authors:** Takumi Suzuki, Nozomu Iwabuchi, Ryosuke Tokuda, Oki Matsumoto, Tetsuya Yoshida, Masanobu Nishikawa, Kensaku Maejima, Shigetou Namba, Yasuyuki Yamaji

**Affiliations:** a Department of Agricultural and Environmental Biology, Graduate School of Agricultural and Life Sciences, The University of Tokyo, Tokyo, Japan; KU Leuven

## Abstract

The complete genome sequence of a pokeweed (Phytolacca americana L.) isolate of mirabilis crinkle mosaic virus (MiCMV) in Japan was determined.

## ANNOUNCEMENT

Mirabilis crinkle mosaic virus (MiCMV) is a single-stranded positive-sense RNA virus recently proposed as a new member of the genus *Potyvirus* (family *Potyviridae*) (1). MiCMV was first isolated from four o’clock (Mirabilis jalapa) plants with mosaic and malformation symptoms on the leaves in China, and three complete genome sequences (GenBank accession numbers MG656405.1, MT247721.1, and MT247722.1) isolated from four o’clocks have previously been reported (1–3). Here, we report the complete genome sequence of MiCMV isolated from pokeweed (Phytolacca americana L.) plants in Japan.

**FIG 1:**
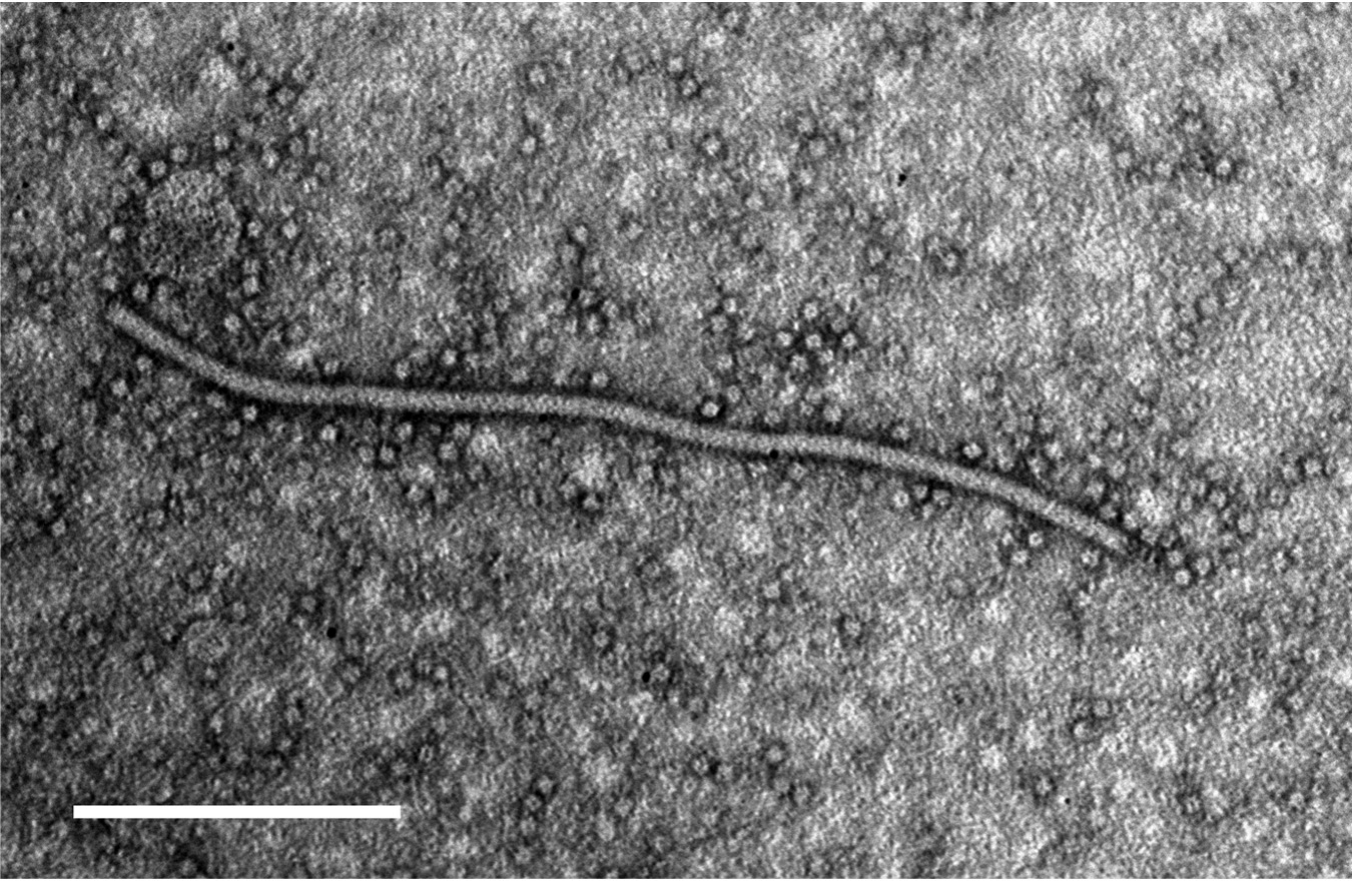
Electron micrograph of a potyvirus-like flexuous filamentous particle observed in crude sap from pokeweed plants with leaf mosaic symptoms stained with 2% phosphotungstic acid. White bar, 200 nm.

In 2019, pokeweed plants with leaf mosaic symptoms were found in Bunkyo Ward, Tokyo, Japan. Electron microscopy observation showed potyvirus-like flexuous filamentous particles, ca. 650 nm long (Fig. 1). Total RNA was extracted from the symptomatic leaves using ISOGEN reagent (Nippon Gene, Japan) and treated with DNase I (TaKaRa, Japan). cDNA was synthesized from the extracted RNA using avian myeloblastosis virus (AMV) reverse transcriptase (Nippon Gene) with a nonadeoxyribonucleotide mixture (TaKaRa). PCR was performed using the potyvirus primers Sprimer (4) and D1000 modified (5) (Table 1). The amplified fragment, approximately 1 kb long, was cloned into a pCR-Blunt II-TOPO vector (Invitrogen, USA) and sequenced using Sanger sequencing. A BLASTN search (6) against GenBank showed that the obtained sequence had 92% identity with the three MiCMV isolates. To determine the complete genome sequence of a single virus isolate from pokeweed plants showing mosaic symptoms, we performed three successive single local lesion transfers in Chenopodium quinoa. Then, a single local lesion isolate was maintained in Nicotiana benthamiana for further assays. Total RNA was extracted from N. benthamiana leaves using an ISOSPIN plant RNA kit (Nippon Gene). cDNA was synthesized from the extracted RNA using AMV reverse transcriptase with GeneRacer oligo(dT) primer (Invitrogen). Then, the entire genome excluding the 5′ end was amplified as three partially overlapping fragments using MiCMV-specific primers designed from the cloned partial sequence and the reported genome sequences (1) and KpGR3nest (7), a specific primer designed on the GeneRacer oligo(dT) primer (Table 1). The 5′ end of the genome was amplified using the 5′ RACE System for Rapid Amplification of cDNA Ends v. 2.0 (Invitrogen). Each fragment was cloned and sequenced as described above. The complete genome sequence was constructed by assembling all overlapping fragments using ATGC v. 4.3.5 (Genetyx, Japan), which showed 100% identity in the overlapping regions.

**TABLE 1:** Primers used in this study

Primer^*a*^	Sequence (5′ to 3′)	Position(s) (bp)^*b*^
RT-PCR
GeneRacer oligo(dT) primer	GCTGTCAACGATACGCTACGTAACGGCATGACAGTGTTTTTTTTTTTTTTTTTT	poly(A)
PCR
Sprimer	GGNAAYAAYAGYGGNCARCC	7945–7964
D1000 modified	GTNCCRTTNTCNATRCACCANAYCAT	8984–8959
1F	AAATTAAAACAACTCATAACAACATACAG	1–29
3kR	CGCAGAAGCACAAACCAAAGTG	3029–3008
3kF	GCAATTAGAACAGGAGTGGCG	2946–2966
6kR	GGAGCAGCTTACACAGAGAAG	5841–5821
6kF	GCAGGGAAGTTTATGGTGATGATGG	5777–5801
KpGR3nest	GGGGTACCGCTACGTAACGGCATGACAGTG	GeneRacer oligo(dT) primer
5′ RACE
GSP1	GGAATGCTCTGATCTGCTCC	541–522
GSP2	GATCCTGCAGCCATTCCTATAGCG	465–442

a RT-PCR, reverse transcriptase PCR; RACE, rapid amplification of cDNA ends.

b Locus based on the complete genome sequence of MiCMV-PA (GenBank accession number LC603132).

The complete sequence of MiCMV isolated from pokeweed (MiCMV-PA) was 9,662 nucleotides (nt) long with 43.1% GC content, excluding the poly(A) tail at its 3′ end. Open reading frame (ORF) prediction using NCBI ORFfinder (https://www.ncbi.nlm.nih.gov/orffinder/) revealed that the genome contained a large ORF (nt 175 to 9417) encoding a putative 350.3-kDa polyprotein. An alignment of the polyprotein amino acid sequence of MiCMV isolates showed that MiCMV-PA had nine polyprotein cleavage motifs conserved in the family *Potyviridae* (8) at the positions corresponding to those of the previously reported MiCMV isolate (1), with only one amino acid variation in the P1/HC-Pro cleavage site (VHHY/S). PIPO (nt 2934 to 3167), a conserved small ORF induced by transcriptional slippage at highly conserved GA_6_ motif among *Potyvirus* species (9, 10), was predicted within the P3 protein-coding region in the −1/+2 reading frame, based on the GA_6_ motif (nt 2929 to 2935). The sequence identities of the large ORF calculated between MiCMV-PA and the three MiCMV isolates using the MUSCLE algorithm (11) in the program SDT v. 1.2 (12) were 90.6% and 94.4% to 94.5% at the nucleotide and amino acid levels, respectively. All tools were run with default parameters. Based on the current species demarcation criteria for the family *Potyviridae*, which have less than 76% nucleotide identity and less than 82% amino acid sequence identity to the large ORF and its protein product, respectively (13), MiCMV-PA was found to belong to the same species as the three other MiCMV isolates.

### Data availability.

The MiCMV-PA genome sequence has been deposited in the DNA Data Bank of Japan/GenBank under accession number LC603132.
